# Capsaicin Changes the Pattern of Brain Rhythms in Sleeping Rats

**DOI:** 10.3390/molecules28124736

**Published:** 2023-06-13

**Authors:** Lei Liu, Yuhua Tian

**Affiliations:** Department of Pharmacology, School of Pharmacy, Qingdao University Medical College, No. 1 Ningde Road, Qingdao 266073, China; liulei0261@163.com

**Keywords:** capsaicin, EEG, TRPV1, brain rhythms, thermoregulation

## Abstract

The heat and capsaicin sensor TRPV1 ion channels were originally discovered in sensory neurons of dorsal root ganglia, and later found in many other tissues and organs. However, whether TRPV1 channels are present in brain regions other than the hypothalamus has been a subject of debate. Here, we addressed this issue with an unbiased functional test by recording electroencephalograms (EEGs) to examine whether capsaicin injection directly into the rat lateral ventricle could alter brain electrical activity. We observed that EEGs during the sleep stage could be significantly perturbed by capsaicin, whereas EEGs during the awake stage did not show a detectable change. Our results are consistent with TRPV1 expression in selective brain regions whose activities are dominative during the sleep stage.

## 1. Introduction

Capsaicin, a natural product derived from chili pepper, is a highly selective agonist for TRPV1 [1]. For example, it does not activate TRPV1 orthologues such as TRPV2, which exhibits a high level of similarities in both linear sequence and 3D structure [2,3]. In TRPV1 knockout mice, treatment with capsaicin cannot induce pain behavior such as paw licking, capsaicin aversion behaviors and change in core body temperature [4]. Therefore, functional evidence of TRPV1 activities induced by capsaicin can serve as a specific and sensitive means to detect TRPV1 expression in selected tissues and organs. The brain is particularly amenable to such an approach for several reasons. It is well-isolated from the rest of the body, allowing the administration of capsaicin selectively to brain tissues. Changes in brain activities can be sensitively and noninvasively detected, using methods such as electroencephalograms (EEGs). Furthermore, brain activities exhibit two clear distinctive phases—awake and sleep—during which different brain regions are active and sensitive to functional perturbation. Recently, the beneficial effects of capsaicin on brain diseases such as Parkinson’s disease, Alzheimer’s disease, stroke and depression have been identified through their animal models. These positive effects are mostly attributed to the presence of TRPV1 channels in the brain [5]. However, a few studies have demonstrated that capsaicin can improve epileptiform activity by inhibiting voltage-gated sodium channels [6,7]. Additionally, besides voltage-gated sodium channels, this pungent compound can also modulate voltage-activated calcium channels in rat dorsal root ganglion neurons [8]. These findings suggest that the effects of capsaicin on different types of channels depend on the applied concentration and specific type of neurons.

The sleep stage of EEGs is triggered by ‘sleep-active neurons’, which are GABA and certain neuropeptide-expressing neurons located in the hypothalamus and thalamus [9]. Sleep is divided into the rapid eye movement sleep (REM sleep) phase and non-rapid eye movement sleep (NREM sleep) phase. NREM sleep is also called slow-wave sleep (SWS) because large slow EEG rhythms such as delta rhythms are dominative in this state. Besides delta waves, theta waves also occur during some sleep stages. The other two types of brain rhythms, alpha and beta waves, are associated with wakefulness and alertness. These brain rhythms are the results of the synchronous activity of many pyramidal neurons in the cerebral cortex and useful for detecting certain pathological conditions such as epileptic seizures and sleep disorders [10,11,12]. Therefore, EEG waves can serve as a sensitive reporter of electrical activities in different brain regions. A considerable amount of research in the literature has demonstrated that ion channels such as T-type Ca^2+^ channels, GABA and glutamate receptors are associated with different sleep states through measuring EEGs of related transgenic animals [9,13,14,15]. Whether TRPV1 participates in shaping the pattern of brain rhythms through modulating ion flow in different brain regions remains unclear.

In the present study, we measured and analyzed the four major EEG rhythms (delta, theta, alpha and beta) in either the awake or sleep stage after the lateral ventricle administration of capsaicin to explore whether TRPV1 is functionally expressed in the brain and whether TRPV1 activation can alter the electrical activities of the brain. Our results demonstrated that capsaicin altered the pattern of EEG rhythms in the sleeping stage but not in the awake stage. These findings indicate that capsaicin can affect EEGs, likely through the presence of TRPV1 channels in the brain, and their distribution is likely uneven across brain regions.

## 2. Results

### 2.1. Capsaicin and Injection Effectiveness Tests

**Figure 1:**
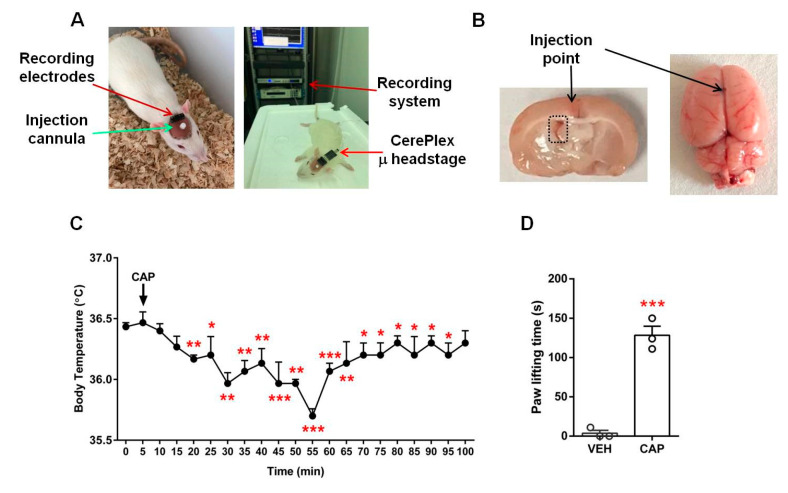
Experimental setup and control experiments. Photographs of the EEG measurement setup (**A**) and the location of the injection point of rat brains (**B**). Black arrows in B indicate the precise injection point of i.c.v injection; the black dashed rectangle in the left panel, image taken 30 min after injection, marks the left lateral ventricle where the injected red dye is visible. (**C**) Changes in body temperature after a single dose of capsaicin (1 mg/kg, subcutaneous; *n* = 3). (**D**) Low-dose capsaicin-evoked hind paw lifting (*n* = 3). * *p* < 0.05, ** *p* < 0.01 and *** *p* < 0.001 versus the initial normal body temperature or vehicle group; paired *t*-test (**C**); unpaired *t*-test (**D**). Data are presented as mean ± SEM. VEH: vehicle, CAP: capsaicin.

To ensure the reliability of the operations for rat brain EEG recordings, we carried out two sets of control tests. First, to ensure that our capsaicin sample was effective for activating TRPV1, we conducted a physiological test by measuring rat body temperature after the subcutaneous injection of capsaicin (1 mg/kg). We observed a rapid decline in body temperature from the stable body temperature prior to injection (36.4 ± 0.03 °C; *n* = 3). At 20 min after capsaicin injection, the body temperature was already significantly lower, at 36.2 ± 0.03 °C (*n* = 3; *p* < 0.01). The body temperature continued to decrease and hit a minimum value at 55 min (35.7 ± 0.06 °C; *n* = 3; *p* < 0.001). The body temperature then gradually returned to the normal level within about 100 min (Figure 1C). The body temperature test confirmed the capsaicin sample we used was effective.

Next, to assess the validity of the selected dose of capsaicin for EEG recordings, we injected a low dose of capsaicin (23 μg/20 μL) into the plantar skin of the rat hind paw. The animals exhibited strong paw lifting responses during the five-minute observation period compared to the control group animals (capsaicin, 128.3 ± 11.5 s; control, 3.7 ± 3.7 s; *n* = 3 each; *p* < 0.001; Figure 1D). As the hind paw tissue likely provided a stronger diffusion barrier than that in the lateral ventricle injection, these behavior results indicate that the selected capsaicin doses were effective for activating TRPV1 under our experimental conditions.

### 2.2. Recording Changes in EEGs upon Brain Injection of Capsaicin

**Figure 2:**
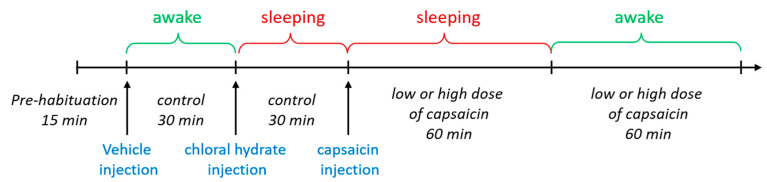
Experimental scheme for EEG recording.

**Figure 3:**
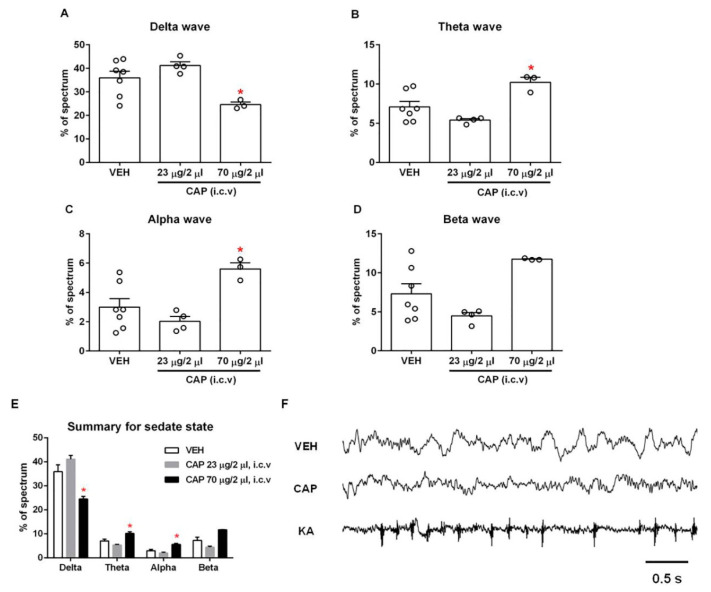
Effect of capsaicin on the sleeping stage EEGs. The delta (**A**), theta (**B**) and alpha (**C**) waves showed a marked change in proportion at a high dose of capsaicin compared to the vehicle group (*n* = 3–7), whereas the beta wave (**D**) did not show a significant change (*n* = 3–7). (**E**) Summary of four types of waves in the sleeping stage. (**F**) Example EEG recordings. Intracerebroventricular capsaicin injection changed the pattern of brain waves in the sleeping stage. Robust spikes appeared in the EEG after kainic acid injection under the same experimental conditions. *, *p* < 0.05 vs. vehicle group; one-way ANOVA followed by Tukey’s multiple comparisons test; data are presented as mean ± SEM. VEH: vehicle, CAP: capsaicin, KA: kainic acid.

EEGs were monitored in both sleeping and awake rats. To ensure the reliability of the experiment, we designed three sets of EEG measurements (Figure 2). We first monitored EEGs upon the injection of just the vehicle, followed by capsaicin injection. We then conducted additional EEG recordings in the same animal after the injection of 1 μg/1 μL kainic acid (KA, an analog of the excitatory amino acid transmitter glutamate used to produce seizures) into the lateral ventricle through the same infusion cannula. Whereas the EEG signals following the vehicle and capsaicin injections were hard to distinguish by eye, prominent seizure spikes after the KA injection were clearly identifiable (Figure 3F), indicating that the brain injection operation was successful. The EEG waveforms from such confirmed individual experiments were analyzed off-line.

### 2.3. Capsaicin Produced a Significant Change in the Pattern of Brain Rhythms in the Sleeping Stage

A delta wave is a high-amplitude brain wave that is defined as having a frequency range of 0.5–4 Hz. It is correlated with the deep sleep stage 3 of NREM sleep. Delta waves have been associated with various neurological disease such as Alzheimer’s disease, epilepsy and schizophrenia [16,17,18]. We observed a significant decrease in the proportion of delta waves in the high-dose capsaicin (70 μg/2 μL)-treated rats compared to vehicle-treated rats (capsaicin, 24.6 ± 1.1%; vehicle, 35.9 ± 2.9%; *n* = 3–7; *p* < 0.05), indicating that TRPV1 activation by capsaicin can affect the deep sleep stage 3 of NREM sleep (Figure 3A). Interestingly, both the theta (4–7.5 Hz) and alpha waves (8–11 Hz) increased significantly after the administration of high-dose capsaicin (theta: 10.2 ± 0.6%; alpha: 5.6 ± 0.4%) compared to the control group injected with vehicle only (theta: 7.1 ± 0.7%; alpha: 3.0 ± 0.6%; *n* = 3–7; *p* < 0.05; Figure 3B,C). Similarly, the proportion of beta waves (15–32 Hz) also exhibited an increase upon high-dose capsaicin injection (capsaicin, 11.7 ± 0.1%; vehicle, 7.3 ± 1.3%; *n* = 3–7; *p* > 0.05), though the change did not reach statistical significance (Figure 3D). In summary, the injection of high-dose capsaicin could substantially affect EEGs in the sleep stage, though individual waves responded differently.

We also tested the effects of low-dose capsaicin (23 μg/2 μL) on EEG in the sleep stage (Figure 3A–D). Whiles low-dose capsaicin injection produced slight changes in all waves, none of these changes reached the level of statistical significance. Figure 3E and Table 1 summarize changes of each brain wave type in the sleeping stage. It can be seen that the low dose and high dose of capsaicin elicited an opposite trend in every type of brain wave. The reason for this interesting phenomenon remains unclear, though one interpretation might be that the low dose of capsaicin was simply insufficient to activate TRPV1-expressing neurons in the brain. Alternatively, it is also possible that the low dose of capsaicin was sufficient to activate the TRPV1 in neurons involved in shaping the sleeping stage EEGs, whereas the strong channel activation caused by the high dose of capsaicin might have led to rapid TRPV1 desensitization that inhibited these neurons. In summary, the observation of significant changes in EEGs suggests that TRPV1 channels are indeed functionally expressed in brain regions that are involved in shaping EEGs in the sleep stage.

### 2.4. Capsaicin Did Not Produce Detectable Changes in the Pattern of Brain Rhythms in the Awake Stage

**Figure 4:**
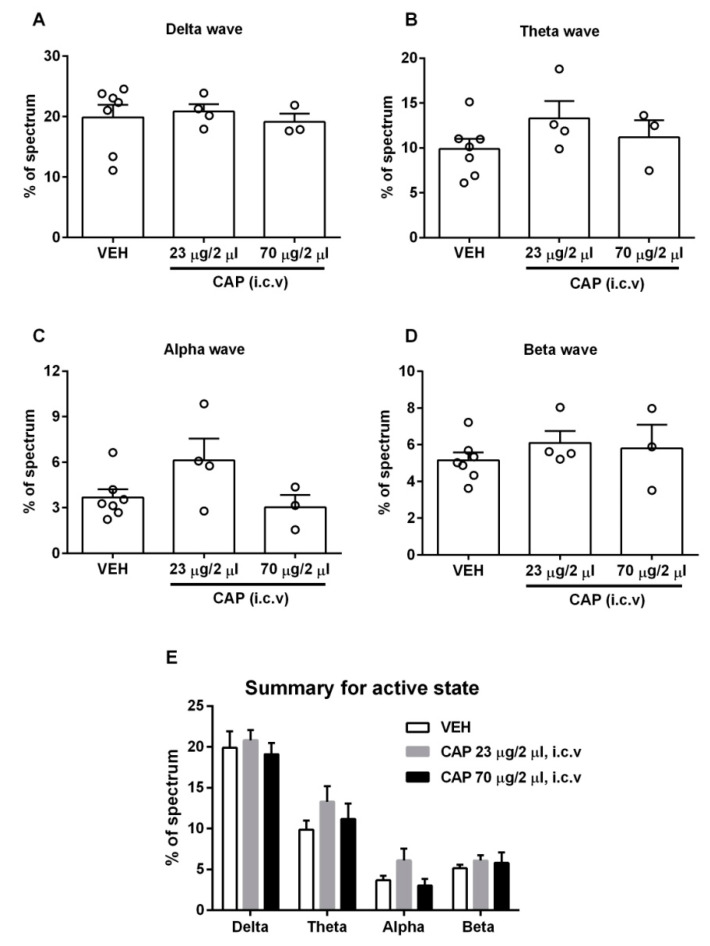
Effect of capsaicin on the awake-stage EEG. (**A**–**D**) The four types of brain waves did not show significant changes in proportion at either dose of capsaicin compared to the vehicle group (*n* = 3–7). (**E**) Summary of four types of waves in the awake state. Data are presented as mean ± SEM. VEH: vehicle, CAP: capsaicin.

In order to collect natural awake brain waves, we conducted EEG recordings in freely moving rats. Unlike the recordings in the anesthetic-induced sleeping stage, none of the brain waves had a statistically significant change in the percentage of the total spectrum (Figure 4). These results demonstrate that capsaicin-induced changes in the pattern of brain waves are state-specific, and capsaicin is only effective for altering the pattern of brain waves in the sleeping stage. While the observations appear to indicate an absence of TRPV1 expression in the brain regions involved, a potential alternative interpretation is that the TRPV1-expressing neurons are already active in the awake stage, making them less sensitive to further excitation.

## 3. Discussion

While EEG measurement offers one of the most direct and sensitive methods of detecting brain responses to capsaicin in live animals under physiological conditions, the approach is by no means trivial. Even though we have confirmed the effectiveness of our injection procedure, there remain several uncertainties that might affect experimental observations. First, the diffusion of the injected capsaicin from the lateral ventricle into various brain regions was not directly observable but is likely to be unequal. The presence of KA-induced seizures added confidence to some degree; however, it is realized that KA is a charged molecule at neutral pH. Capsaicin, which is of a similar molecular size to KA, is highly lipophilic, a property that may facilitate tissue penetration but at the same time allow trapping. Second, as discussed earlier, the activation of TRPV1 is transient, with prolonged stimulations quickly leading to channel desensitization. Third, an individual neuron’s response to capsaicin is dependent not only on TRPV1 expression but also on its excitation state. A deeply dormant neuron would require a stronger stimulus to fire action potentials; on the other hand, an already highly active neuron may be less sensitive to excitatory stimuli. Given these concerns, it is likely that positive results from the EEG measurement would be simpler to interpret than negative results.

**Figure 5:**
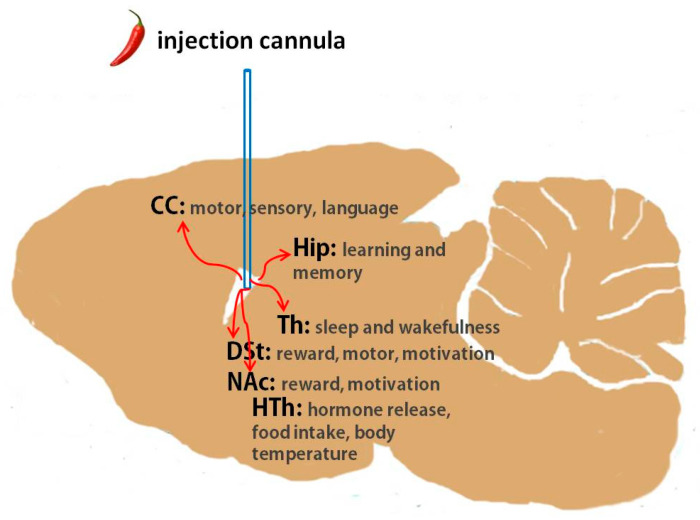
Rat brain diagram illustrating the location of capsaicin injection in relation to the cerebral cortex, hippocampus, dorsal striatum, nucleus accumbens, thalamus and hypothalamus. CC: cerebral cortex, Hip: hippocampus, DSt: dorsal striatum, NAc: nucleus accumbens, Th: thalamus, HTh: hypothalamus.

What are the brain regions likely participating in the EEG changes that we observed? Figure 5 illustrates the location of capsaicin injection in a rat brain, with the regions discussed below highlighted. A number of studies have documented that the hypothalamus is a critical brain region for regulating sleep and wakefulness as well as for shaping EEGs. Importantly, previous research has found TRPV1 expression in the hypothalamus, which is involved in regulating food intake and body temperature [19,20,21,22]. Therefore, it is perhaps not surprising that capsaicin injection may affect EEGs through activating TRPV1 in the hypothalamus. Two brain regions where the detection of TRPV1 expression has previously been reported [23,24,25], DSt and NAc, are in close proximity to the source of injected capsaicin, and hence it is likely that these regions were activated during our tests. Both DSt and NAc are known to participate in the regulation of slow-wave sleep [26,27]. The hippocampus, another site with reported TRPV1 expression [28,29,30,31], is also very close to the injection site and is known to contribute to EEGs during non-rapid eye movement (NREM) sleep [32,33,34]. The cerebral cortex contains a wide variety of neurons, and EEGs summarize the broad patterns of excitatory and inhibitory post-synaptic potentials from the dendrites of the pyramidal neurons of this cortex [35]; hence, the cerebral cortex is a potential region that may have been perturbated by capsaicin in our study. Additional sites, for example, the thalamus, a critical region for the dual control of sleep and awake stages [36,37,38,39], might also potentially be involved based on previous studies demonstrating TRPV1 distribution in the thalamus [40,41].

While our claim regarding the role of TRPV1 in the changes in EEGs induced by capsaicin is supported by our EEGs, our current in vivo experiments alone do not provide direct evidence that TRPV1 is the only ion channel affected by capsaicin. This is due to the lack of convincing results from in vitro and additional animal tests. Therefore, to obtain more conclusive conclusions, it is important to conduct further experiments, such as immunohistochemistry after capsaicin EEG recordings and the inclusion of antagonists such as capsazepine in future capsaicin EEG recordings. Furthermore, since our doses of capsaicin were relatively high and may not induce a highly selective effect on TRPV1 channels, we cannot exclude the possibility that capsaicin may affect the sleep stage through other channels, such as voltage-gated sodium channels [6,42].

In summary, our EEG recording data have indicated the correlation between direct TRPV1 activation and brain rhythms. However, further experiments are necessary to confirm the exact function of TRPV1 in EEGs. By utilizing this unbiased functional test, we have provided strong evidence for the possibility of TRPV1 expression in the rat brain. The information should guide future investigations of the roles TRPV1 may play in higher brain functions.

## 4. Materials and Methods

### 4.1. Chemicals

Capsaicin was purchased from Abcam (Cambridge, UK). All other chemicals not specified in the results section were obtained from Sigma (St. Louis, MO, USA).

### 4.2. Animals

Male wild-type Sprague Dawley rats (Beijing Vital River Laboratory Animal Technology Co., Ltd., Beijing, China) weighing 190–210 g (6 weeks old) were housed one per cage for a minimum of three days before experimental use and fed rat chow and water ad libitum. The experimental protocols were approved by the Animal Use and Care Committee of Qingdao University.

### 4.3. Control Experiments

To validate the activity of our capsaicin sample, we conducted two sets of control tests. We measured body temperature using an infrared electronic thermometer (TRULY, Shanwei, China) every 5 min after capsaicin (1 mg/kg) subcutaneous (s.c.) injection for 100 min. We also carried out a behavioral test evaluating the time an animal spent lifting the hind paw after it was injected with capsaicin, following reported procedures [4,43].

### 4.4. Implant Procedure

Rats were placed on stereotaxic apparatus, anaesthetized with 400 mg/kg of chloral hydrate intraperitoneally (i.p.) and received (s.c.) a local anesthetic agent, lidocaine (1.5 mg/kg), on the top of the head. Five EEG screw electrodes were implanted into the skull. Four screws (active electrodes) were implanted either near the dorsal striatum (DSt: AP (anteroposterior) = 2.0 mm anterior to the bregma, ML (mediolateral) = ±2.5 mm to the bregma) or near the hippocampus (Hip: AP = 3.0 mm posterior to the bregma, ML = ±3.0 mm to the bregma). The fifth screw was implanted in the cerebellum as a reference electrode. For intracerebroventricular (i.c.v.) microinjections, a stainless steel guide cannula (RWD Life Science, Shenzhen, China) was implanted into the lateral ventricle of the brain, using the following coordinates: AP = 0.8 mm posterior to the bregma, ML = 1.5 mm lateral to the bregma, DV (dorsoventral) = 3.8 mm below the surface of the skull. The EEG electrodes and guide cannula were then secured with dental cement. The free ends of the electric wires that were connected to the screws were soldered to pins in a socket that was mounted onto the skull with dental cement (Figure 1A, left).

### 4.5. EEG Recording and Analysis

Seven days after surgery, each rat was gently restrained, and an infusion cannula (RWD Life Science, Shenzhen, China) was inserted into the lateral ventricle through the guide cannula to a depth of 3.8 mm below the surface of the skull. A vehicle or capsaicin solution with a volume of 2 μL was infused into the lateral ventricle in a 2 min period followed by an additional 1 min to allow diffusion before the injection needle was removed. The socket on the rat brain was connected to a CerePlex µ headstage of the EEG recording system through flexible recording cables on the day of EEG recording (Figure 1A, right). EEGs were recorded using a CerePlex^TM^ Direct system (Blackrock microsystems, Salt Lake City, UT, USA; sampling rate, 1 kb/s; bandpass, 0–250 Hz) (Figure 1A, right). We recorded EEG patterns in both the sleeping and awake stage. For EEG recording in the sleep stage, we anaesthetized each rat using chloral hydrate (400 mg/kg, i.p.) and put it on a Styrofoam board at stable room temperature (25 °C) to prevent a steep decline in body temperature. For EEG recording in the awake stage, each rat was placed in a cage that permitted free movement. Baseline EEG recording was performed for 15 min before vehicle injection. Subsequently, an EEG was recorded continuously in the awake and sleeping stage, first after vehicle treatment for a 30 min period and then after capsaicin injection for a 1 h period. At the beginning of the experiment, a total of ten rats were initially included. However, during the adaptation period in the recording chamber, three out of ten rats removed the cannula themselves, rendering them unable to undergo EEG measurements. As a results, only seven rats remained for the experiment. Then, we injected the vehicle into the rats. After finishing baseline EEG recording in the awake and sleeping states, each for 30 min, the rats were divided into two groups: four rats for a low dose of capsaicin and three rats for a high dose of capsaicin. Subsequently, EEG recordings were conducted in the sleep and awake states for each rat for 1 h (Figure 2). As depicted in the scheme, the entire experimental procedure took over three hours to continuously record EEGs in different states. To analyze various sleep waves, we specifically selected data from the first 30 min of each stage. These data were then normalized to the control group of the respective state, allowing us to obtain a relative percentage of each wave type. Additionally, it should be highlighted that the hypnotic effects of chloral hydrate can be controlled by adjusting the drug administration interval. During the EEG recording period, we also measured the animal’s body temperature every 2 min. Digitized data were transmitted to a computer and analyzed with NeuroExplorer version 5 (Nex Technologies, Colorado Springs, CO, USA). Fast Fourier transformation (FFT) analysis of data collected after capsaicin treatments was used for the analysis of qualitative sleep parameters such as delta (0.5–4 Hz), theta (4–7.5 Hz), alpha (8–11 Hz) and beta (15–32 Hz) power spectra. After the EEG recordings were completed, we removed the entire brain to confirm the location of i.c.v. injection (Figure 1B).

### 4.6. Statistical Analysis

For comparison, one-way ANOVA followed by Tukey’s multiple comparisons test were used for data obtained from EEG recordings; a paired *t*-test was used for body temperature measurement data; an unpaired *t*-test was used for the pain behavior test. Statistical significance was set at at least *p* < 0.05. Statistical data are expressed as mean ± SEM.

## Figures and Tables

**Table 1:** Summary of the proportion of each brain wave in total spectrum in the sleeping stage after capsaicin injection (*n* = 3–7).

	Delta Wave	Theta Wave	Alpha Wave	Beta Wave
vehicle	35.94 ± 2.85%	7.07 ± 0.70%	2.99 ± 0.59%	7.30 ± 1.29%
23 μg/2 μL capsaicin	41.14 ± 1.56%	5.39 ± 0.19%	2.02 ± 0.33%	4.47 ± 0.45%
70 μg/2 μL capsaicin	24.56 ± 1.09% ^$^	10.21 ± 0.64% ^#^	5.60 ± 0.42% ^&^	11.74 ± 0.07%

^$^ *p* < 0.05 vs. the percentage of delta wave of vehicle group; ^#^ *p* < 0.05 vs. the percentage of theta wave of vehicle group; ^&^ *p* < 0.05 vs. the percentage of alpha wave of vehicle group. One-way ANOVA followed by Tukey’s post-test.

## Data Availability

Data is contained within the available article.

## Sample Availability

Samples of the compounds are available from the authors.

## Abbreviations

TRPV1	Transient receptor potential cation channel, subfamily V, member 1
EEG	Electroencephalogram
